# The Clinical Frailty Scale as useful tool in patients with brain metastases

**DOI:** 10.1007/s11060-022-04008-5

**Published:** 2022-04-13

**Authors:** Johannes Kerschbaumer, Aleksandrs Krigers, Matthias Demetz, Daniel Pinggera, Julia Klingenschmid, Nadine Pichler, Claudius Thomé, Christian F. Freyschlag

**Affiliations:** Department of Neurosurgery, Medical University of Innsbruck, Anichstr. 35, 6020 Innsbruck, Austria

**Keywords:** Frailty, Karnofsky, Performance, Score, Brain metastasis, Neurooncological, Surgery

## Abstract

**Purpose:**

The Clinical Frailty Scale (CFS) evaluates patients’ level of frailty on a scale from 1 to 9 and is commonly used in geriatric medicine, intensive care and orthopedics. The aim of our study was to reveal whether the CFS allows a reliable prediction of overall survival (OS) in patients after surgical treatment of brain metastases (BM) compared to the Karnofsky Performance Score (KPS).

**Methods:**

Patients operated for BM were included. CFS and KPS were retrospectively assessed pre- and postoperatively and at follow-up 3–6 months after resection.

**Results:**

205 patients with a follow-up of 22.8 months (95% CI 18.4–27.1) were evaluated. CFS showed a median of 3 (“managing well”; IqR 2–4) at all 3 assessment-points. Median KPS was 80 preoperatively (IqR 80–90) and 90 postoperatively (IqR 80–100) as well as at follow-up after 3–6 months. CFS correlated with KPS both preoperatively (r = − 0.92; p < 0.001), postoperatively (r = − 0.85; p < 0.001) and at follow-up (r = − 0.93; p < 0.001).

The CFS predicted the expected reduction of OS more reliably than the KPS at all 3 assessments. A one-point increase (worsening) of the preoperative CFS translated into a 30% additional hazard to decease (HR 1.30, 95% CI 1.15–1.46; p < 0.001). A one-point increase in postoperative and at follow-up CFS represents a 39% (HR 1.39, 95% CI 1.25–1.54; p < 0.001) and of 42% risk (HR 1.42, 95% CI 1.27–1.59; p < 0.001).

**Conclusion:**

The CFS is a feasible, simple and reliable scoring system in patients undergoing resection of brain metastasis. The CFS 3–6 months after surgery specifies the expected OS more accurately than the KPS.

## Introduction

Brain metastases often cause rapid neurological deterioration in patients that already suffer from systemic tumor symptoms [1–3]. Chemotherapy or radiation therapy can lead to severely limited general well-being and additional neurological symptoms like deterioration in motor function, seizures or language problems, which are associated with hospitalization [4–6].

Radiation therapy is the primary treatment applied in the majority of cases [7, 8], mainly as radiosurgical intervention as most classical systemic cancer treatments do not perform well in the protected niche of the central nervous system (CNS) [9, 10]. In cases with multiple metastases or after surgical resection, palliative whole brain radiotherapy (WBRT) has been proposed as standard treatment. Surgery has shown advantages only in particular cases with limited numbers of brain metastases [11–13], although recent data suggests that surgery also plays a role in multiple metastases [14].

Neurosurgeons face an increasing number of patients with brain metastases, as prolonged systemic tumor control with novel cancer drugs increases overall survival [15].

Unfortunately, treatment options for brain metastases have not evolved in the same way. Systemic agents able to enter the CNS are reserved to certain entities only, like melanoma or non-small-cell lung cancer (NSCLC) with driver mutations, but cannot be applied to the vast majority of brain metastases (BM) [16]. Radiotherapy (RT) and surgery are often the only options. Advances in radiotherapy have changed the use of palliative WBRT towards radiosurgical interventions [17] with the goal to preserve functional brain tissue and decrease cognitive decline [18].

These treatments are suitable for small to medium size metastases, but large metastases with mass effect and neurological deficits due to compression are poorly treated by radiotherapy [13, 19, 20]. Surgical excision of symptomatic BMs leads to the fast relief of symptoms and diminishes the need of corticosteroids. Surgery can also account for prolonged overall survival (OS) [21–23].

Contemporary management of BM patients is an individual concept that consists of systemic treatment, focused radiotherapy and surgical resection of selected metastases [13].

In order to identify patients that benefit from surgical intervention, different scores have been applied. The Karnofsky Performance Score (KPS) is a widely used, quickly applicable score in medical and neurological oncology [24, 25]. However, it has been introduced to evaluate the patient’s suitability to undergo chemotherapy or radiation therapy and withstand the treatments’ toxicity and adverse reactions, rather than as an accurate tool for outcome prediction.

For brain metastases, more accurate scores like the Graded Prognostic Assessment (GPA) index have been introduced to estimate these patients’ survival [26, 27]. Although the KPS has been updated with diagnosis-specific score items for different entities [28], these scores are not easily applicable and therefore not routinely used.

The term “frailty” describes an individual’s risk to become dependent or even die when exposed to a stressor and is widely used in geriatric medicine [29]. In intensive care medicine, frailty is an independent predictor of long-term mortality [30]. In other surgical specialties like orthopedics, it is associated with increased need of revision surgery and concomitant morbidity [31].

In 2005, Rockwood et al. developed the CFS for geriatric patients (see Table 1), but it has been rarely applied in neuro-oncology [32, 33].

**Table 1:** The Clinical Frailty Scale (CFS) according to Rockwood et al.

1 *Very Fit*—People who are robust, active, energetic and motivated. These people commonly exercise regularly. They are among the fittest for their age	6 *Moderately Frail*—People need help with *all outside activities* and with *keeping house*. Inside, they often have problems with stairs and need *help with bathing* and might need minimal assistance (cuing, standby) with dressing)
2 *Well*—People who have *no active disease symptoms* but are less fit than category 1. Often, they exercise or are very *active occasionally*, e.g. seasonally	7 *Severely Frail*—*Completely dependent for personal care*, from whatever cause (physical or cognitive). Even so, they seem stable and not at high risk of dying (within 6 months)
3 *Managing Well*—People whose *medical problems are well controlled*, but are *not regularly active* beyond routine walking	8 *Very Severely Frail*—Completely dependent, approaching the end of life. Typically, they could not recover even from a minor illness
4 *Vulnerable*—While *not dependent* on others for daily help, often *symptoms limit activities.* A common complaint is being “slowed up”, and/or being tired during the day	9 *Terminal Ill*—Approaching the end of life. This category applies to people with a *life expectancy < 6 months,* who are *not otherwise evident frail*
5 *Mildly Frail*—These people often have *more evident slowing*, and need help inn *high order IADLs* (finances, transportation, heavy housework, medications). Typically, mild frailty progressively impairs shopping and walking outside alone, meal preparation and housword	

The aim of this study was to use the Clinical Frailty Score (CFS) in surgical patients harboring brain metastases in order to quickly assess a patient’s general condition and to decide whether a patient qualifies for a neurosurgical intervention. The results are compared to the more frequently used KPS.

## Materials and methods

Patients who underwent surgical resection of 1–3 BM in one session at our department between 2005 and 2019 were selected from our neuro-oncological database and retrospectively assessed applying KPS and CFS. Clinical and demographic data was retrieved from our electronic database, if available and correlated to the clinical course of their disease.

KPS and CFS was rated before the surgical intervention and upon discharge as well as 3–6 months after the surgery. According to Rockwood et al. [32] The KPS was evaluated prospectively by the clinician during the routine examination as an internal standard of care. CFS was assessed retrospectively by one author only disclosed to the patients’ clinical presentation.

Additional variables delivered from the database were primary tumor origin, number of brain metastases resected, the extent of resection assessed on postoperative MRI (within 48 h), surgeons’ estimated extent of resection, surgeons experience (trained or in training) and cerebral progression on MRI according to RANO criteria [34].

Statistical analysis and graphics were processed using IBM SPSS Statistics (IBM SPSS Statistics for Mac OS, Version 27.0. Armonk, NY: IBM Corp.), Graphpad prism 9 for Mac OS (GraphPad Software, San Diego, California) and Adobe Photoshop for Mac OS 22.3.0 (Adobe Inc., USA). The magnitude of association between KPS and CFS as non-parametric data was evaluated with Spearman correlation tests and corrected according to Holm-Bonferroni processing for multiple hypothesis. Kaplan Meier curves, dichotomizing good Clinical Frailty Score (1–4) and poor CFS (5–9) according to log-rank processing were shown. Hazard ratios for death were calculated for independent parameters using Cox regression analysis. p values < 0.05 were considered as statistically significant.

The conducted trial was approved by the ethics committee of the Medical University of Innsbruck (1333/2021) and the investigation was performed in accordance with the ethical standards of the 1975 Declaration of Helsinki, as amended in 2013.

## Results

### Demographics

A total of 205 patients (110 male and 95 female) aged between 18 and 85 years (median 61 years) were included in this study. Primary tumors were distributed within common incidences with NSCLC being the most frequent (44.9%), followed by breast cancer and melanoma (11.2% and 14.1% respectively, see Table 2).

**Table 2:** Demographics

No. total	205
Gender, n(%)	
Male	110 (53.7)
Female	95 (46.3)
Primary, n(%)	
Lung (NSCLC)	92 (44.9)
Breast	23 (11.2)
Melanoma	29 (14.1)
RCC	5 (2.4)
CUP	6 (2.9)
Others	50 (20)
Location, n(%)	
Supratentorial	170 (82.9)
Infratentorial	35 (17.1)
Eloquent	52 (25.4)
GTR, n(%)	
Surgeon-estimated	194 (94.6)
MRI-defined	159 (77.6)

*NSCLC* non-small-cell lung cancer, *RCC *renal cell cancer*, CUP* cancer of unknown primary, *GTR* gross total resection

Eighty-three percent of the resected metastases were located supratentorially versus 17.1% in the cerebellum. 74.6% of the BM occupied non-eloquent areas of the brain.

A complete resection could be achieved in 77.6% according to early postoperative MRI, with an expected discrepancy to the surgeons’ intraoperative rating of gross total resection (GTR) of 94.6%.

Only a small proportion of the patients showed transient postoperative worsening of their neurological status (12.7%, n = 26), whereas 7.8% (n = 17) improved in neurological status immediately after surgery and 79.5% (n = 163) were found to be stable.

Median follow-up (FU) of the included patients amounted to 10 months (IqR 4–25). Patients who died during the follow-up were followed for a median of 7 months (IqR 7–16) and patients that were still alive at time of analysis had a median follow-up of 62 months (IqR 4–25).

### Frailty

Preoperatively, the patients demonstrated a median CFS of 3 (“managing well”, IqR 2–4) that could be preserved postoperatively and during the 3–6 months follow-up.

KPS was 80 preoperatively (median, IqR 80–90) and increased to 90 (median, IqR 80–90) postoperatively, remaining stable at the follow-up (IqR 80–100) at 3 to 6 months.

**Fig. 1:**
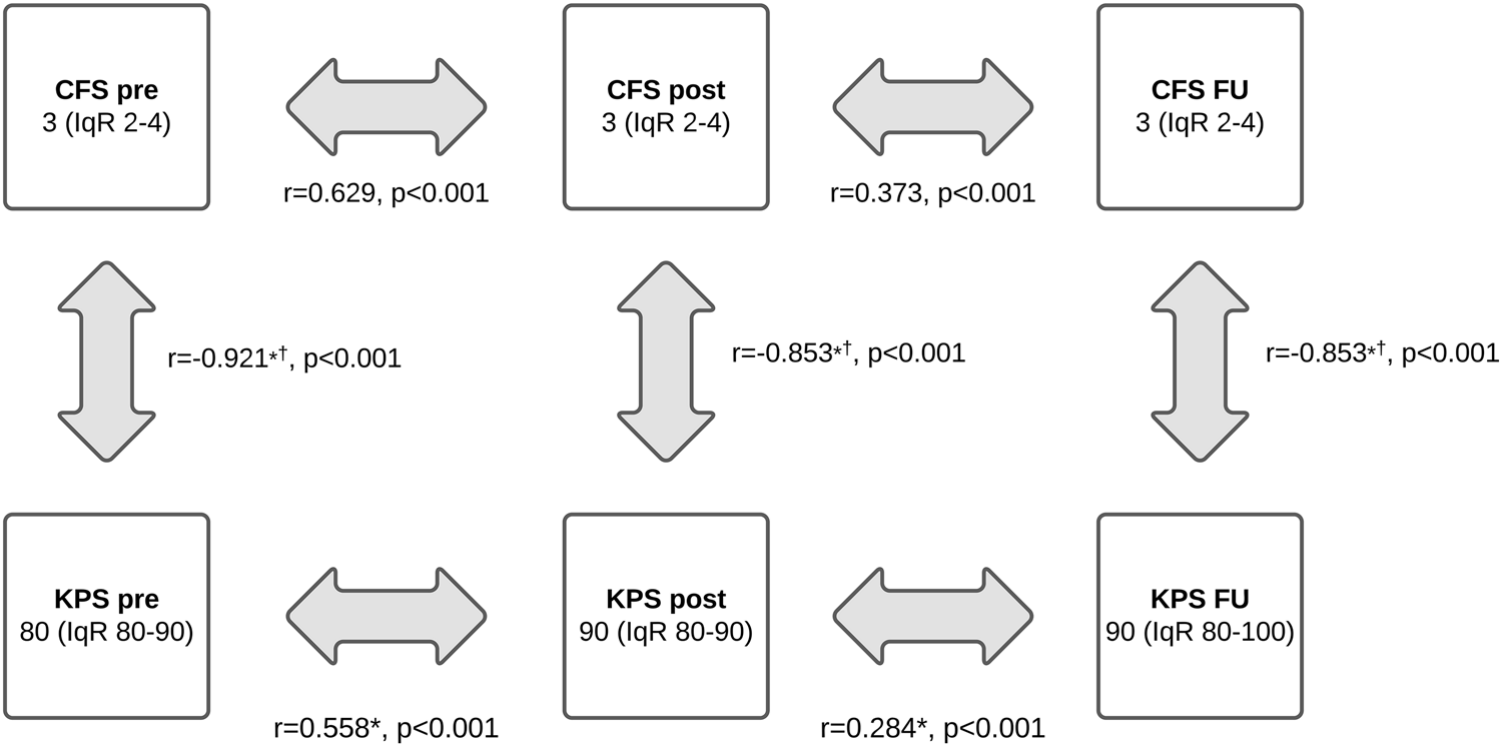
The Rockwood Clinical Frailty Scale (CFS) and the Karnofsky Performance Score (KPS) preoperatively (pre), postoperatively (post) and at 3–6 months postoperatively (FU), shown as median and interquartile range (IqR). ^*^Correlation is significant at the 0.001 level (2-tailed). ^■^Negative correlation because of better performance with higher KPS but lower CFS

The preoperative CFS correlated significantly with the postoperative CFS (p < 0.001) and the score at the 3–6 months follow-up (p < 0.001). Spearmen’s test revealed a moderate correlation between pre- and postoperative CFS (r = 0.629) and a weak correlation of preoperative CFS and the performance at the 3–6 months follow-up (r = 0.309; see Fig. 1).

Monovariate analysis revealed a lower preoperative CFS of male patients with a median CFS of 4 (IqR 3–4) compared to a CFS of 3 (IqR 2–4) in women (p = 0.026). Postoperatively, a comparable performance (male: female–3:2 [both IqR 2–4], p > 0.05.) was noticed and in the 3–6 months follow-up male patients performed worse with a median CFS of 3 (IqR 2–4) compared to a CFS of 2 (IqR 1–4) in female patients (p = 0.047).

Neither the number of resected BM (1–3) nor the experience level of the surgeon (resident vs. consultant) nor the location of the metastasis showed a significant influence on the CFS. Only BM in eloquent locations were associated with lower CFS and KPS preoperatively (CFS/KPS: p = 0.008/p = 0.007) and postoperatively (p = 0.009/p = 0.008), but this correlation was lost at follow-up. If a new neurological deficit was recorded after surgery, CFS and KPS scores worsened significantly (p < 0.001).

**Fig. 2:**
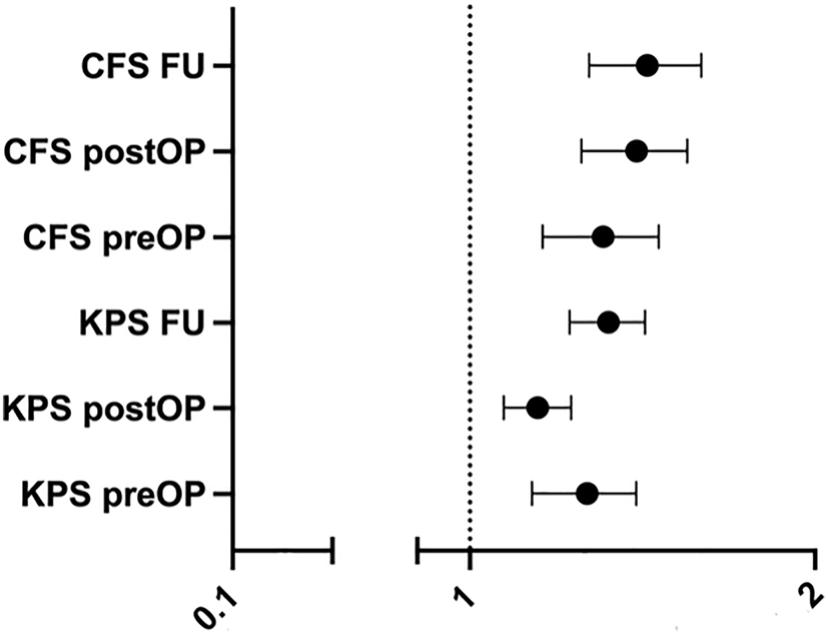
Hazard ratios (HR) for Clinical Frailty Scale (CFS) and Karnofsky Performance Score (KPS) with 95% CI. HR for KPS was inverted, because higher KPS resembles a better performance, as lower CFS does also

The Cox regression analysis revealed a significant influence of the KPS on OS preoperatively (HR 1.267 per 10 point-step, CI 1.132–1.395), postoperatively (HR 1.142 per 10 point-step, CI 1.070–1.225, p < 0.001) and at 3–6 months FU (HR 1.320 per 10 point-step, 1.221–1.420, p < 0.001). The CFS demonstrated an even stronger prediction of outcome preoperatively (HR 1.3 per step, CI 1.157–1.460, p < 0.001), postoperatively (HR 1.394 per step, CI 1.258–1.545, p < 0.001) and at 3–6 months follow-up (HR 1.421 per step, CI 1.270–1.590, p < 0.001) (see Fig. 2).

In a Cox regression model of the data with KPS and CFS entered pairwise, CFS was superior at predicting the clinical course postoperatively (CFS HR 1.527 [CI 1.234–1.890], p < 0.0001; KSP—n.s.; model p < 0.0001) and 3–6 months FU (CFS HR 1.288 [CI 1.051–1.578], p = 0.014; KSP—n.s.; model p < 0.0001), as well as trended preoperatively (CFS HR 1.292 [CI 0.974–1.715], p = 0.076; KSP—n.s.; model p < 0.0001).

The predictor for the clinical course in the Cox regression model of all performance scores with “enter” processing was the CFS at the 3–6 months FU (HR 1.247 [CI 1.020–1.523], p = 0.031; other—n.s.; model p < 0.0001).

Additionally to CFS postoperatively, patients’ age showed a significant impact on the clinical course with a HR of 1.029 (p < 0.001) as well as the preoperative tumor volume (HR 1.013, p = 0.004) and the number of resected brain metastases (HR 1.240, p = 0.007).

**Fig. 3:**
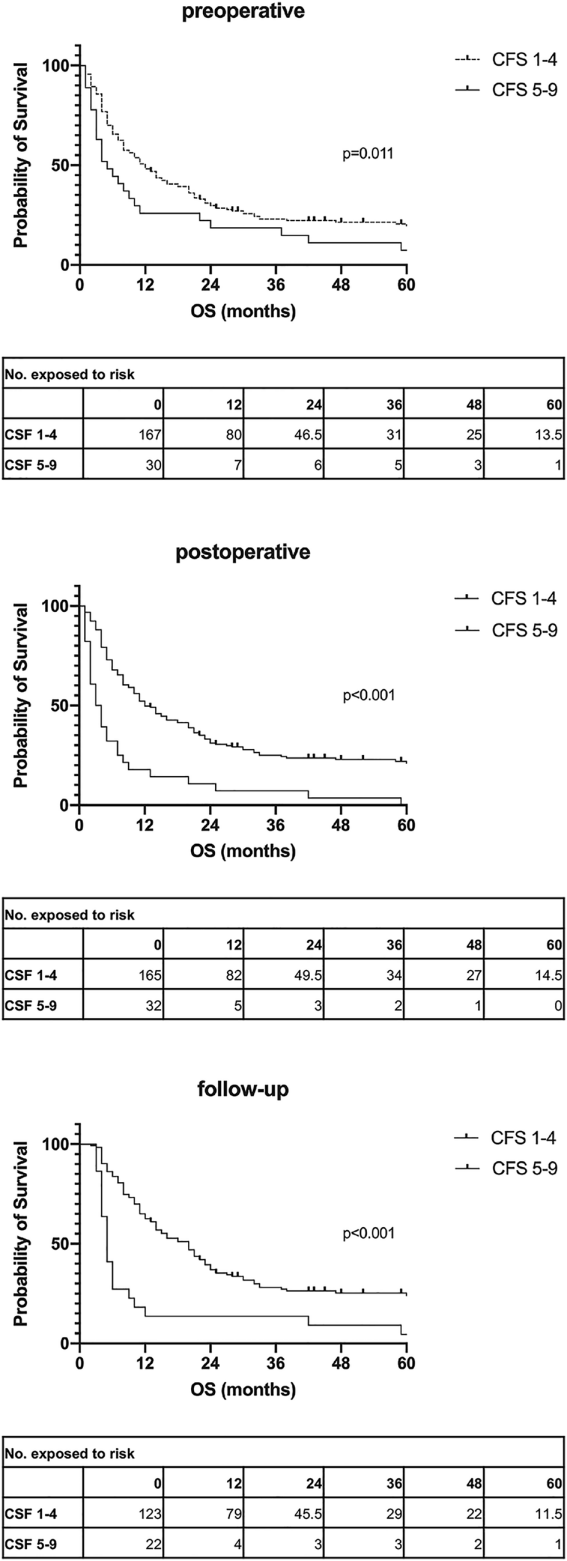
Kaplan Meier curves, dichotomizing good Clinical Frailty Score (CFS 1–4) and poor CFS (5–9) according to log-rank processing with number exposed to risk for the two groups. Median survival time accounted for 11 (95% CI 7.277–14.723) months and 4 (95% CI 1.322–6.678) months for the good CFS group and poor CFS group preoperatively, 12 (95% CI 9.015–14.985) months and 3 (95% CI 1.778–4.222) months postoperatively and 20 (95% CI 15.399–24.601) months and 5 (95% CI 4.096–5.904) months at 3–6 months postoperatively

When conducting a Kaplan Meier survival analysis, patients with good Clinical Frailty Score (CFS 1–4) clearly had a longer overall survival than those with poor CFS (5–9) according to log-rank processing (see Fig. 3).

## Discussion

According to our data, the Clinical Frailty Score (CFS) can serve as a reliable score for clinical performance of patients with BM and further as a predictor for OS. The CFS was a significantly superior predictor than the KPS. Especially the CFS at 3–6 months follow-up showed a statistically highly significant prognostic value concerning overall survival. Due to the symptomatic burden of BMs, the initial preoperative CFS can be worsened and showed only limited influence (r = 0.3) on postoperative CFS. This indicated that even patients with poor CFS upon presentation can improve considerably after surgical resection. The CFS could thus become a helpful postoperative tool in neuro-oncology.

Surgical resection can be in patients with intracranial metastases, but is primarily applied for large lesions with mass effect or extensive edema. Prognosis of the clinical course and OS are difficult to determine, since multiple different factors need to be considered. This includes not only extracranial tumor response, but also the number of brain metastases [35–37]. Previous studies on the prognosis and outcome of patients harboring BM from different primary tumors have considered various clinical parameters and scores but not a frailty index. The Clinical Frailty Score is a key part of assessment in geriatric medicine, but is not yet routinely used in neuro-oncology. The widely used KPS has only limited predictive value for the OS and does not cover all relevant aspects, like previous medical conditions. We herein suggest the use of CFS that can quickly be applied and provides a more reliable prognostic tool in neuro-oncological patients.

The overall median preoperative CFS of 3 in our cohort indicates that the patients are “managing well” and suggests that these patients are able to care for themselves. This status could be preserved postoperatively as well as at the 3–6-month follow-up in our cohort. Therefore, surgical resection did not negatively influence the patients’ postoperative well-being and general condition. This finding is of fundamental importance, given the often palliative setting and aim for the surgeon to not impair the patients’ quality of life (QoL). A low risk for postoperative morbidity in patients with BM has already been shown in the literature and was confirmed in this study [38–40]. Furthermore, in our cohort CFS, KPS, and neurological examination improved postoperatively or at the 3–6 months follow-up, again arguing against a decline in the patients' quality of life due to the surgical intervention.

Also, patients with BM in eloquent areas of the brain seem to profit from surgical resection. Even if they present with a low CFS and KPS pre- and postoperatively, the results do not differ from those of patients with “non-eloquent” BM at 3–6 months FU. Clearly, space-demanding BM within the primary motoric cortex or other eloquent centers in the CNS lead to neurological alteration early-on, given the usually large surrounding tissue reaction of BM [41]. Even if that does not account for an immediate relief of symptoms, shown with the CFS postoperative, the patients recover quickly [21, 42] and seem comparable to patients with metastases in non-eloquent areas. Any new permanent neurological deficits need to be avoided, even cases with high symptomatic burden, where recovery will take some time, because permanent deficits lead to decline in CFS and KPS dramatically (p < 0.001). This has a direct influence on the patients’ quality of life and results in shorter OS [39, 43, 44].

Age was also a significant predictor of outcome in this analysis. As reported in the literature, elderly patients have an increased risk for a poor prognosis, which was confirmed in this study [40, 45]. However, the increased preoperative frailty could also have an influence, since elderly patients are more likely to present with higher CFS and worse performance scores. Not only the higher risk of comorbidities like cardiopulmonary disease and increased fragility of blood vessels could play a role [40], but also worse tolerance to chemotherapies and could cause a poorer outcome in older patients. These aspects will gain importance in coming years with an aging population in industrialized countries. Elderly patients with a good preoperative CFS can achieve a good postoperative outcome, as shown in our analysis, what could be of clinical relevance. Thus, the decision regarding further therapy and possible resection should not only be based on age, but moved towards the patients’ condition and frailty.

Since the CFS is quickly assessable but seems to be of higher predictive value than the KPS, the authors recommend its use for a more integrated view of patient prognosis.

## Limitations

The limitations of our study are that a single-center-cohort might not be representative and there may be a selection bias, because we only included surgically treated patients. A confirmation in other data sets is needed to confirm the importance of this score in patients with brain metastases. The retrospective study design has to be mentioned, possibly affecting the assessment of neurological status, the CFS and the KPS. We compared exclusively the universal clinical scores and did not integrate disease-specific scores like GPA. We tried to overcome this by generously excluding patients with insufficient documentation of their neurological status.

## Conclusion

This study investigates the CFS as a novel score to predict the clinical course in patients with brain metastases. The decision on treatment of patients should be based on an integrative analysis, considering all available parameters. Age was shown to be a risk factor for worse prognosis, but CFS should be considered independently, as elderly patients with good CFS may as well experience a good outcome. Given that patients with BM often present with neurological deficits and a quick decision concerning further therapy is mandatory, the CFS could be a helpful tool in decision-making.

## Data Availability

The datasets generated during and/or analyzed during the current study are available from the corresponding author on reasonable request due to privacy and ethical restrictions.
